# Low Rates of Early Aseptic Loosening of the Cemented Attune S+ Total Knee Arthroplasty System

**DOI:** 10.7759/cureus.88406

**Published:** 2025-07-21

**Authors:** McKenna Sheridan, Pranav Krishnan, Hassan Farooq, Whisper Grayson, Harold Rees, Nicholas Brown

**Affiliations:** 1 Orthopaedic Surgery, Loyola University Stritch School of Medicine, Maywood, USA; 2 Orthopaedic Surgery & Rehabilitation, Loyola University Medical Center, Maywood, USA

**Keywords:** aseptic loosening, attune total knee system, cemented total knee arthroplasty, complications, total knee arthroplasty (tka)

## Abstract

Background

Aseptic loosening is a common cause of late revision surgery performed following total knee arthroplasty (TKA). Recent advances in implant designs have aimed at reducing this risk, with the cemented Attune S+ total knee system focused on improving stability through enhanced cement adherence. Despite excellent preliminary in vitro data, there is a lack of clinical studies assessing the performance of this implant, with prior studies reporting high rates of aseptic loosening. The primary objective of this study was to assess the clinical outcomes, primarily rates of aseptic loosening and revision, of Attune S+ implants.

Methodology

This retrospective study included 47 patients who underwent TKA using the cemented Attune S+ system between November 2017 and April 2023. All surgeries were performed by a single fellowship-trained arthroplasty surgeon at a tertiary care academic medical center. The primary outcome was aseptic loosening, which was evaluated on postoperative knee radiographs for stress shielding and radiolucent lines.

Results

There were no patients with radiographic evidence of aseptic loosening, and no known revision surgeries were performed. Of note, one patient had a subsequent operation 817 days after the original TKA to remove scar tissue in the knee joint due to arthrofibrosis, but no signs of prosthetic failure were found intraoperatively.

Conclusions

In this study, there were no cases of aseptic loosening or revision surgery observed with the cemented Attune S+ TKA system.

## Introduction

Total knee arthroplasty (TKA) remains a successful treatment option for patients with end-stage knee osteoarthritis [1]. While failure after TKA remains an infrequent occurrence, aseptic loosening accounts for up to 40% of all late revision surgeries, occurring at least two years after the index procedure [2-5]. As a result, efforts have focused on improving prosthesis design to decrease complication rates and improve patient outcomes.

The Attune total knee system (DePuy Synthes), first introduced in 2012, aimed to increase stability and implant survivorship with a novel prosthetic curvature, a comprehensive range of sizes, and a newly designed polyethylene insert [6]. While early studies detailed positive findings relating to pain and functional scores, reports emerged detailing the failure of the prosthesis at the tibial implant-cement interface [7-10]. Attributed to factors ranging from increased constraint of the tibial component, altered cement binding properties, and surgeon inexperience, the lack of prosthetic fixation caused unintended micromotion and resultant aseptic loosening [8-10].

The modified Attune S+ system was developed in 2017 to address this potential complication [11]. Macrolock pockets were added on the inferior aspect of the tibial stem to promote cement interdigitation [11]. Additionally, surface roughness of the prosthesis was increased to allow for improved cement adherence [11]. While preliminary in vitro studies have demonstrated the increased binding properties of this new prosthesis, there is minimal published data on its clinical performance [12]. The purpose of this study was to investigate the rate of aseptic loosening with Attune S+ implants in a single surgeon’s practice at a tertiary care academic medical center. We hypothesize a low rate of aseptic loosening in the short-term postoperative period with the Attune S+ system.

## Materials and methods

Patient selection and variables of interest

Following institutional review board approval (approval number: 217339), a retrospective chart review of a single surgeon’s patients undergoing TKA was performed. Inclusion criteria included patients ≥18 years of age who had undergone a cemented TKA using the cemented Attune S+ system between November 24th, 2017, and April 1st, 2023, for primary osteoarthritis. Exclusion criteria included revision surgeries, cementless implants, and non-Attune prostheses. After patient identification, patient demographics and medical comorbidities (Charlson Comorbidity Score) were recorded, while postoperative clinic notes were consulted for signs of prosthesis failure, including complaints of pain, limited mobility, and prosthesis instability. Knee radiographs were evaluated for signs of postoperative complications, including stress shielding (defined as bone resorption adjacent to the tibial base plate) and radiolucent lines by a single reviewer. No minimum follow-up period was required for inclusion.

Operative technique

All surgeries were performed by one board-certified, adult reconstruction, fellowship-trained surgeon. Patients underwent general anesthesia before the operation. A standard midline incision with a medial parapatellar approach was used. In total, 29 operations underwent mechanical alignment while the other 18 underwent kinematic alignment with an unrestricted caliper technique [13,14]. All patients underwent appropriate tibial baseplate sizing and were taken through full ranges of motion to ensure adequate seating. All baseplates were cemented with knees in extension and adequately irrigated. Both Attune PS and CR implants were included in this study.

## Results

Patients

A total of 47 patients who underwent a primary TKA with the cemented Attune S+ system between November 2017 and April 2023 were identified. No patients were lost to follow-up or censored. A majority of the patients (83%) were female, with an overall average age of 67 years (range = 42 to 85 years) at the time of the TKA. The average Charlson Comorbidity Index was 4 (SD = 3.0) (Table 1).

**Table 1:** Baseline demographics of the study population (n = 47). Means and SDs are provided for continuous variables. Percentages and sample sizes are provided for categorical variables. TKA = total knee arthroplasty

	Study cohort (n = 47)
Average Age at TKA (years)	67.0 (10.5)
Average time without complications post-TKA (months)	21.6 (16.6)
Gender	
Female	83.0% (39)
Male	17.0% (8)
Race/Ethnicity	
Caucasian	55.3% (26)
African American	34.0% (16)
Hispanic	4.3% (2)
Other/Unknown	6.4% (3)
Average Charlson Comorbidity Index	4.0 (3)

Outcomes following total knee arthroplasty

None of the patients had any clinical signs of aseptic loosening during postoperative visits. The average amount of time post-TKA without any clinical signs of complications was 21.6 months, with an SD of 16.6 months (of note, one patient died less than one year following their TKA). None of the patients had any radiographic evidence of aseptic loosening (stress shielding or radiolucent lines) on their most up-to-date knee radiographs. Lastly, none of the 47 patients have had a known TKA revision. Of note, one patient had a subsequent operation 27 months after the original TKA to remove scar tissue in the knee joint due to arthrofibrosis, but no signs of prosthetic failure were found intraoperatively.

## Discussion

The primary aim of this study was to assess the rate of early aseptic loosening in patients who underwent TKA using the cemented Attune S+ system. In this study, among the 47 patients followed for a mean of 21.6 months post-surgery, none demonstrated clinical or radiographic evidence of aseptic loosening, and no patients required revision surgery. This is a promising finding, especially considering previous reports on the Attune system, which highlighted concerns about high rates of tibial baseplate loosening, a leading cause of TKA failure [3-5].

One of the most notable aspects of our study is the absence of aseptic loosening in the cohort, despite a follow-up period that averaged nearly two years. The absence of radiolucent lines or signs of stress shielding in the radiographs further supports the notion that the Attune S+ system is performing well in the short to medium term. This contrasts with the high rates of loosening reported in earlier studies of the Attune system. For instance, Bonutti et al. documented 13 cases of aseptic loosening requiring revision surgeries across multiple centers, and Murphy et al. described two cases of tibial loosening, one requiring bilateral revisions [4,5]. These reports raised concerns regarding the long-term performance of the original Attune system, particularly related to tibial baseplate fixation. However, the Attune S+ system, which features modifications such as Macrolock cement pockets and a roughened baseplate surface, appears to have yielded a more favorable outcome in our cohort. The design changes in the Attune S+ system likely contributed to its improved fixation, which may explain the absence of loosening in this study, in line with the findings of Jaeger’s in vitro research on cement debonding, where the Attune S+ showed superior resistance to failure compared to the original Attune system [7].

Moreover, our findings are consistent with national registry data of the Attune TKA system. According to the American Academy of Orthopaedic Surgeons’ American Joint Replacement Registry Annual Report, the usage of the Attune system has dramatically increased between 2012 and 2023 [15]. Attune accounts for 20.8% of all TKAs performed on patients 65 years or older with a diagnosis of primary osteoarthritis [15]. Of the 92,489 Attune TKAs performed between 2012 and 2023, only 1.9% required revisions after 10 years [15]. While our study showed zero subjects requiring revisions due to aseptic loosening, national registry data still shows a very low percentage of Attune TKA patients requiring a revision. In addition, this registry data accounts for revisions performed for all indications, suggesting revision rates due to aseptic loosening are even lower than what is accounted for with this data.

Interestingly, while the average follow-up duration of 21.6 months is relatively short in the context of TKA longevity (which typically spans decades), it is sufficient to capture early signs of aseptic loosening or failure. The absence of these signs in our cohort provides valuable insights into the early effectiveness of the Attune S+ system. Longer follow-up periods are necessary to fully assess the long-term durability of this prosthesis, but our data suggest that the Attune S+ system does not present an unusually high rate of early loosening.

The demographic characteristics of our study population reflect a cohort typical of TKA patients. The average Charlson Comorbidity Index of 4 further highlights the presence of significant comorbid conditions in the sample, which may influence both surgical outcomes and recovery. Although the cohort was relatively heterogeneous, there were no evident disparities in the incidence of loosening or revision between different patient groups. Future studies with larger sample sizes may provide further insights into how specific demographic or comorbidity factors may influence outcomes with the Attune S+ system.

There are several limitations to this study that should be acknowledged. First, the retrospective design and relatively small sample size limit the generalizability of the findings. A larger, multicenter prospective study would provide more robust data regarding the performance of the Attune S+ system across different patient populations and surgical settings. Additionally, a comparative group would further strengthen the study. While all of the radiographic measurements were performed by the same person to limit potential variability, this introduces the risk of intra-observer errors. Additionally, although no cases of aseptic loosening or revision surgery were identified during the study period, the follow-up duration was not long enough to rule out the possibility of loosening occurring later. Long-term follow-up is crucial to assess whether the Attune S+ system maintains its stability over time. The inclusion of both PS and CR implants introduces another potential limitation. The biomechanics of CR and PS components differ primarily in how they manage posterior stability. These differences can lead to varying patterns of load transmission, which may influence stress distribution at the bone-cement interface, potentially affecting implant fixation and long-term cement integrity. Nevertheless, no aseptic loosening was witnessed with either implant. Lastly, another limitation is the lack of functional outcome measures, such as the Knee Society Score or Western Ontario and McMaster Universities Osteoarthritis Index, which would have provided a more objective measurement of the clinical benefits of the Attune S+ system. Future registry-based prospective studies should incorporate these measures to better assess the success of the implant in terms of both patient-reported outcomes and functional recovery.

## Conclusions

Our study suggests that the cemented Attune S+ TKA system demonstrates a low rate of early aseptic loosening, with no cases of loosening or revision surgery observed in a cohort followed for up to three years. These findings provide preliminary evidence that the design modifications incorporated into the Attune S+ system, particularly the enhanced cement fixation features, may significantly reduce the risk of early loosening. However, further studies with longer follow-up periods and larger sample sizes are necessary to fully evaluate the long-term durability and clinical outcomes of the Attune S+ system.
